# Pediatric Oncology Knowledge Mobilization in Canada: A Environmental Scan

**DOI:** 10.1177/00469580261468769

**Published:** 2026-07-15

**Authors:** Catherine Foulem, Emily K. Drake, Angelina Lui, Sharon Dias, Ekaterini Damoulianos, Stephanie Reid, Patrick Cossette, Michel Duval, Kirsten Efremov, James Foster, Karen Haas, Argerie Tsimicalis

**Affiliations:** 1 Gerald Bronfman Department of Oncology, Faculty of Medicine and Health Sciences, 5620 McGill University, Montréal, QC, Canada; 2 Ingram School of Nursing, Faculty of Medicine and Health Sciences, 5620 McGill University, Montréal, QC, Canada; 3 Interdisciplinary Health Studies, Faculty of Science, 289915 Mount Allison University, Sackville, NB, Canada; 4 Department of Surgical and Interventional Sciences, Faculty of Medicine and Health Sciences, 5620 McGill University, Montréal, QC, Canada; 5 Department of Social Work, 725810 Trent University Durham-GTA, Oshawa, ON, Canada; 6 Lived Experience Researcher, Toronto, ON, Canada; 7 Leucan, Montréal, QC, Canada; 8 Centre de Recherche, 25461 Centre Hospitalier Universitaire Sainte-Justine, Montréal, QC, Canada; 9 Département de Pédiatrie, 5622 Université de Montréal, Montréal, QC, Canada; 10 Kids Cancer Care, Calgary, AB, Canada; 11 Lived Experience Researcher, Oro-Medonte, ON, Canada

**Keywords:** knowledge mobilization, cancer, children, teens, pediatric, non-profit

## Abstract

**Introduction:**

Non-profit organizations play a key role in supporting knowledge dissemination activities. However, their contribution to these activities in-person and online in the pediatric oncology field remains unclear. Our study aims to provide an overview of the pediatric oncology charitable organizational landscape and describe their knowledge mobilization efforts related to dissemination, highlighting existing strengths, gaps, and opportunities to strengthen and unite efforts.

**Methods:**

A bilingual environmental scan was conducted using an internet-based search strategy to identify Canadian pediatric oncology non-profit organizations. Eligible organizations were screened, and data on their characteristics and knowledge dissemination strategies were extracted. Extracted data were analyzed descriptively and presented in tables, spatially, and narratively with figures.

**Results:**

The scan identified 906 results, and 25 organizations were eligible for inclusion in the study. Eleven organizations (44%) are based in Ontario. The decade where the highest number of organizations were founded is 2000–2009 (n = 9, 36%), offering primarily English-only content (n = 18, 72%). Only six (26.1%) provided bilingual resources. Among the knowledge dissemination strategies, digital knowledge dissemination activities were utilized often, with all organizations using websites and social media (particularly Facebook, n=25 (100%), and Instagram, n =24 (96%)).

**Conclusion:**

This study identified the current list and knowledge dissemination efforts of non-profit organizations in the pediatric oncology community in Canada but reveals gaps in bilingual content and in other major languages spoken in the country, geographic physical location, and centralized resources.

## Introduction

In Canada, about 1000 children from birth to the age of 14,^1^ and about 400 adolescents between the age of 15 to 19 years old are diagnosed with cancer each year.^2^ Of those diagnosed with pediatric cancers, 84% reach the five-year survival milestone and this number is expected to increase with new treatments.^3^ However, pediatric cancers may pose significant financial and personal challenges for families, as parents and caregivers often need to dedicate considerable time and resources to treatment and to supporting their children.^4^ Moreover, among the children with a disease duration of 3-4 years, 25% may experience difficulties in peer relationships, and 27% may feel isolated.^5^ The need for sharing credible information is crucial for all of those involved in the pediatric cancer community (e.g. patients, families, clinicians, academia, scientists, etc.). Collectively, they can help to prevent the spread of misinformation.^6,7^ With the correct information, caregivers can make informed decisions and children and teenagers can better understand the health matters that concern their life and, therefore, be included in conversations and decisions with caregivers.^8,9^ Parents often struggle to understand the information provided to them, which can result in misunderstandings and mistrust.^8^ Furthermore, although some parents prefer that filtered information is conveyed to their children, pediatric patients stated that they want to be informed about their own diagnosis and prognosis,^8^ and actively involved in their health care.^10^ Thus, distributing evidence-based information to the broader pediatric oncology community is essential to provide community members with credible resources.^11^ Non-profit organizations play a key role in addressing the gap between information and young patients and their support system through mobilizing, sharing, and disseminating knowledge.

Knowledge mobilization (KMb) refers to the transfer and exchange of research knowledge among researchers, knowledge brokers, and knowledge users.^12,13^ It involves applying evidence-based research to practice and policy.^12,14^ According to Canada’s Social Sciences and Humanities Research Council (SSHRC), knowledge mobilization enables all citizens to benefit from research, enhancing its impact.^15^ Knowledge dissemination, one of the knowledge mobilization activities, is the active process of communicating results to a particular audience.^16^ The targeted distribution of information can expand the reach of evidence and facilitate policy changes based on findings of up-to-date research.^11^ Among the strategies for knowledge dissemination are online resources, which serve as a means to convey information to a broad audience and to involve them in the act of engaging with new knowledge.^17^ For newly diagnosed oncology patients and their families, an educational delivery method they may prefer is the Internet.^18^ However, health literacy, defined as the ability to access and comprehend health information, can influence how families seek knowledge. Individuals with lower health literacy are more likely to seek important information from social media and the Internet.^19^ Hence, the key role of knowledge dissemination through online platforms. Given all the struggles the community and families involved in oncology care endure, and the need for those working in the field to access and share reputable information, there is a need to share evidence-based information from credible organizations. This can be done through non-profit organizations’ websites, social media, and other online platforms.

In Canada, non-profit organizations play a significant role in providing resources for the pediatric oncology community nationwide, offering reliable information that the population trusts.^20,21^ Non-profit organizations often act as knowledge brokers, the liaison between researchers and knowledge users.^16^ These organizations represent a potential mechanism for high levels of social capital, social cohesion, as well as strong informal networks, aiding the community as a whole.^21^ For instance, individuals from various backgrounds and roles in pediatric oncology in Canada have developed and composed ACCESS (Advancing Childhood Cancer Experience, Science & Survivorship), a pan-Canadian network, to achieve better health outcomes and quality of life for children diagnosed with cancer.^22^ ACCESS aims to act as the expert voice of pediatric cancer care and research in Canada, and aims to ultimately transform lives by advancing pediatric cancer experiences, science and survivorship.^22^ Within this context, our goal was to identify all the pediatric oncology non-profit organizations in Canada, and identify how these non-profit organizations disseminate knowledge to the pediatric oncology community. More specifically, the objectives of this environmental scan were to: (1) identify a current list of non-profit organizations serving the pediatric oncology community in Canada; (2) explore and describe how these organizations disseminate information; (3) assess the strengths and gaps in knowledge mobilization, as well as future directions and opportunities for these efforts in Canada; and (4) outline a methodology for conducting an environmental scan using various internet-based sources to engage the pediatric oncology community by combining and utilizing an updated framework for scoping reviews,^23^ and environmental scans methodologies.^24-27^ Our goal was to aid in advancing, supporting, and uniting the strengths of the pediatric cancer community in their ongoing knowledge mobilization efforts in Canada.

## Methodology

Our environmental scan was conducted to systematically map, collect and analyze the information derived from non-profit organizations.^24-26^ The study was led by the team appointed in the Ingram School of Nursing and Gerald Bronfman Department of Oncology at McGill University, in collaboration with members appointed in other universities and non-profits, and/or affiliated with the ACCESS network. The study was conducted in Canada over a one-year period from January 2025 to December 2025, retrieving publicly available online sources of information. Environmental scans serve as an important tool to inform decision-making on policy, planning and program development in the healthcare sector.^28^ We followed the STROBE guidelines for reporting our study.^29^ To develop our methodology,^27^ we utilized the updated framework for scoping reviews by Peters et al^23^ as well as the environmental scan methodology developed by the works of Castro et al,^24^ Choo,^25^ and Rowel et al.^26^ The protocol^27^ was tailored to this new context, to accurately map non-profit organizations and identify their knowledge dissemination efforts in pediatric oncology. Our methodology consisted of: (1) conducting a comprehensive search of databases for non-profit, government, and charitable organizations; (2) screening the results for study eligibility; (3) extracting data from eligible sources; (4) consulting with experts and community; (5) analysing the data; and (6) producing a final report.^24,27^

### Eligibility Criteria

Under the Canadian government, there are distinct definitions.^30^ Non-profit organizations cannot be a registered charity, but there are registered charities referring to themselves as non-profits (e.g., some identify as non-profit organizations with charitable status).^27^ To account for the interchangeable use of these terms at both the governmental and community levels, the term “non-profit” in our study referred to organizations that hold a registered charitable number.^27^

To qualify for inclusion in our environmental scan, organizations had to satisfy the following criteria: (1) be a non-profit organization; (2) be based in Canada; (3) disseminate information and/or resources; and (4) serve the pediatric oncology community such as patients, caregivers, healthcare providers, scientists and/or trainees.^27^ Throughout the development of the protocol,^27^ the terms “non-profit,” “not-for-profit,” and “charitable organizations” were found to often be used interchangeably in both grey literature and the community.^27^ Organizations without a registered charitable number or with missions that target the general childhood health community (e.g., pediatric pain) or broader cancer community (e.g., organizations serving all ages affected by cancer) were excluded from this study. Non-profits that focused on the broad pediatric cancer community or on a specific type of childhood cancer were included.^27^ Due to the paucity of organizations in Canada serving the adolescent and young adult cancer community (15-39 years of age),^31^ those that serve this population were included. We excluded all non-profit organizations serving the adult community (40 years and older). Organizations that serve adults were excluded. Additionally, advocacy groups that do not hold a registered charitable number and/or primarily function as social media influencers or digital opinion leaders were also excluded from this study.^27^ The knowledge dissemination efforts of non-profit organizations were screened to analyze their efforts. Activities were included if they: (1) involved a method of online or in-person knowledge dissemination; (2) were relevant to the pediatric oncology community in Canada; and (3) focused on content that relate to the full spectrum of cancer care delivery.^27^

### Data Sources and Search Strategy

#### Overview

A four-step, bilingual (English and French) search was conducted to identify Canadian non-profit organizations involved in pediatric oncology activities. First, pediatric oncology non-profit organizations were identified by: (1) searching the currently listed partners of a national government-funded network (ACCESS; Advancing Childhood Cancer Experience, Science & Survivorship) for pediatric oncology and the non-profit organizations identified through its development; (2) reviewing three Canadian governmental databases; (3) searching a non-profit, non-governmental, resource database; and (4) consulting experts in the pediatric oncology community and ACCESS members. The development of the search strategy was a collaborative process that was developed by the authors of the protocol,^27^ reviewed by an expert patient/librarian and persons with lived experiences, and then was re-reviewed by the research team members with expertise in pediatric oncology.^24^ This collaboration ensured the keywords selected for the search strategy were effective and aligned with how advocates, people who work in the field and/or persons with lived experiences would seek evidence-based non-profit resources in pediatric oncology. The keywords developed for the search strategy included “pediatric cancer,” “childhood cancer,” and “pediatric oncology,” along with other appropriate translations (see Table 1).^27^ Each keyword was entered individually in the search engine. The data were retrieved from June to July 2025.

**Table 1.** Keywords Developed to Guide the Search Strategy Pertaining to the Objectives. English Terms Were Translated Into French by a Bilingual Co-Author (E.D.)^26^

Original English keywords	Translated French keywords
Childhood cancer	Cancer chez l’enfant
Pediatric cancer	Cancer pédiatrique
Pediatric	Pédiatrique
Pediatric oncology	Oncologie pédiatrique
Child cancer	Cancer de l’enfant
Adolescent cancer	Cancer de l’adolescent
AYA	AJA
Infant cancer	Cancer du nourrisson

#### Step 1: Search of Online Resources and Databases

***Identifying Non-Profit ACCESS Partners.*** First, we searched the non-profit organizations identified through the creation of ACCESS.^32^ This included the organizations identified in 2022 through the CIHR’s^33^ call for the Pediatric Cancer Consortium funding opportunity.^27,32^

***Government of Canada’s Federal Corporation Database*.** The Government of Canada’s Federal Corporation database^34^ was searched using the key terms listed in Table 1. They were entered in the fillable section for corporate names, while the corporate and business number fields remained blank. For the search, the province of the registered office was set to “Any,” and the corporation status remained “Active.” The “Canada Not-for-Profit Corporation Act” was selected as the governing legislation.^27^

***Canada’s Business Registries***. A search was conducted of the federal-provincial-territorial collaborative registry Canada’s Business Registries,^35^ which is supported by the Canadian Association of Corporate Law Administrators.^27^ We conducted the search using the keywords from Table 1. The selections made for location, business type, and business status were “All location”, “All”, and “Active”, respectively. The results were sorted by “Best Match”.^27^

***Government of Canada’s List of Charities and Certain Other Qualified Donees*.** We used the basic search function to search the Canadian federal government’s list of charities and certain other qualified donees.^36^ The key terms from Table 1 were entered for “organization name,” and “registered” was selected for “status.”^27^

***Canadian Cancer Society’s Community Services Locator.*** We searched the directory of the Canadian Cancer Society’s Community Services Locator using the basic search function.^27,37^ Each separate search term was entered in the fillable section for “organization name.” For this search, we did not specify locations or postal codes. The final search results were organized by relevance, and no specific limitations were applied.^24,27^

#### Step 2: Screening

The search results were uploaded to a shared Excel file where duplicates were removed. Each result was assessed against the study’s inclusion criteria to screen for eligibility.^27^ Search results that did not meet the inclusion criteria were excluded. For organizations where eligibility remained unclear, a team discussion was held to determine inclusion or exclusion.^24,27^

#### Step 3: Data Collection

Non-profit organizations that were eligible for inclusion were reviewed by one member of our study team (C.F), who extracted the data (see Table 2). A data extraction template was developed by our team, building on the work of Cooper, Rodway, and Read^15^ to help maintain consistency in the data extraction process.^27^ Data were extracted pertaining to organization name, year founded, registered charitable number, province of headquarters, whether it had a physical office, targeted audience, language of content, and knowledge dissemination strategies.^27^ The data were collected through exploring the labelling of sections on the organizations’ websites and the content that they share on these pages. The knowledge dissemination activities posted on these websites were also extracted (e.g., social media accounts, videos, pamphlets, infographics, newsletters, clinical practice guidelines, training sessions, blogs, and reports).^27^ The results were reviewed by members of the research team.

**Table 2.** Data Extraction Table for Identified Non-profit Organizations in Pediatric Oncology and Their Location and Audience, Language and Method of Dissemination

Organization (Year founded, charity reference number)	Location of head quarters (physical office (yes/no))	Audience for the disseminated information and language of content	Method of dissemination
AYA CAN - Canadian Cancer Advocacy (2023, 774516009 RR 0001)	Manitoba (No)	Patients, Caregivers, Healthcare providers, Scientists, Siblings of patients and English and French	Social media (X, LinkedIn, Facebook, Instagram) Newsletter, Training Session, Virtual information session, Podcast, Academic publication, Webcast, Book, Community call
Camp Quality (1990, 133423962 RR 0001)	Ontario (Yes)	Patients, Caregivers, Healthcare providers, Siblings of patients and English and French	Social media (X, LinkedIn, Facebook, Instagram, Youtube), Blog, Pamphlet, Infographic, Newsletter, Poster, Virtual information session, Report, In-person
Campfire Circle (1989, 131116022 RR 0001)	Ontario (Yes)	Patients, Caregivers, Siblings of patients and English	Social media (X, LinkedIn, Facebook, Instagram, Youtube), Video, Blog, Newsletter, Virtual information session, Report, In-person
Candlelighters - Newfoundland and Labrador (1982, 119220168 RR 0001)	Newfoundland & Labrador (Yes)	Patients, Caregivers, Siblings of patients and English	Social media (X, Facebook, Instagram), Report, In-person
Candlelighters Simcoe (2009, 856292867 RR 0001)	Ontario (Yes)	Patients, Caregivers, Siblings of patients, Teachers, Decision makers and English	Social media (X, Facebook, Instagram), Video, Blog, Newsletter, Training session, Game, Book, In-person
Candlelighters’ Childhood Cancer Support Group (2010, 834124067 RR 0001)	Manitoba (No; PO Box)	Patients, Caregivers, Siblings of patients and English	Social media (Facebook, Instagram), Blog, Newsletter, In-person
Childcan (1975, 118851930 RR 0001)	Ontario (Yes)	Patients, Caregivers, Healthcare providers, Scientists, Siblings of patients and English	Social media (LinkedIn, Facebook, Instagram), Video, Blog, Academic publication, reports
Childhood Cancer Canada (2006, 828252346 RR 0001)	Ontario (Yes)	Patients, Caregivers, Healthcare providers, Scientists, Siblings of patients, English and French	Social media (LinkedIn, Facebook, Instagram), Video, Blog, Infographic, Newsletter, Report, Book
Childhood Cancer Family Support Society (2004, 895308807 RR 0001)	British Columbia (Yes)	Caregivers and English	Social media (Facebook, Instagram), Video
Fight Like Mason Foundation (2017, 706020492 RR 0001)	Ontario (Yes)	Patients, Caregivers, Scientists and English	Social media (X, Facebook, Instagram, Youtube), Video, Blog, Infographic, Podcast, News article, In-person
Fondation Charles-Bruneau (1990, 131903874 RR 0001)	Québec (Yes)	Patients, Caregivers, Healthcare providers, Scientists and English and French	Social media (X, LinkedIn, Facebook, Instagram, Youtube), Video, Blog, Newsletter, Report, In-person
Island Kids Cancer Association (2017, 739636694 RR 0001)	British Columbia (Yes)	Patients, Caregivers, Siblings of patients and English	Social media (Facebook, Instagram), Video, Virtual Information Session, Podcast, In-person
Kids Cancer Care (1994, 899409171 RR 0001)	Alberta (Yes)	Patients, Caregivers, Siblings of patients, Healthcare providers, Teachers and English	Social media (LinkedIn, Facebook, Instagram, Youtube), Video, Blog, Infographic, Newsletter, In-person, Academic publication
Kids with Cancer Society (1987, 886401397 RR 0001)	Alberta (Yes)	Patients, Caregivers, Siblings of patients, Healthcare providers, Scientists, Teacher and English	Social media (Facebook, Instagram), Blog, Webinar, Report, In-person
Leucan (1978, 119018703 RR 0001)	Québec (Yes)	Patients, Caregivers, Siblings of patients, Scientists, Teacher and English and French	Social media (LinkedIn, Facebook, Instagram, Youtube), Blog, Newsletter, Book, CD, DVD, In-person, Report
Northern Ontario Families of Children with Cancer (NOFCC) (2002, 864676713 RR 0001)	Ontario (Yes)	Patients, Caregivers, Siblings of patients and English	Social media (Facebook), Blog, Newsletter, In-person
Ontario Parents Advocating for Children with Cancer (OPACC) (2006, 846324168 RR 0001)	Ontario (Yes)	Patients, Caregivers, Siblings of patients and English	Social media (X, Facebook, Instagram, Youtube), Blog, Infographic, Virtual information session, Report, In-person
Pediatric Oncology Group of Ontario (POGO) (2003, 871067245 RR 0001)	Ontario (Yes)	Patients, Caregivers, Siblings of patients, Healthcare providers, Scientists, Teachers, Indigenous communities, Decision makers and English	Social media (X, LinkedIn, Facebook, Instagram, Youtube), Video, Blog, Newsletter, Clinical practice guideline, Report, In-person, Academic publication
Sophia Smiles Pediatric Cancer Foundation (2024, 767727555 RR 0001)	Nova Scotia (N; PO Box)	Patients, Caregivers and English	Social media (Facebook, Instagram), Blog
Tali’s Fund (2019, 715663282 RR 0001)	Ontario (Yes)	Patients, Caregivers, Siblings of patients, Scientists and English	Social media (LinkedIn, Facebook, Instagram, Youtube), Video, Blog, Newsletter, Virtual information session, In-person, Academic publication
On the Tip of the Toes (2000, 867322844 RR 0001)	Québec (Yes)	Patients, Caregivers, Scientists and English and French	Social media (LinkedIn, Facebook, Instagram, Youtube, Flickr), Blog, Newsletter, In-person, Infographic, Report, Academic publication
The Voboc Foundation (2002, 864166533 RR 0001)	Québec (Yes)	Patients, Caregivers, Healthcare providers and English and French	Social media (LinkedIn, Facebook, Instagram, Youtube), Video, Newsletter, In-person
West Coast Kids Cancer Foundation (2018, 721885291 RR 0001)	British Columbia (Yes)	Patients, Caregivers, Siblings of patients and English	Social media (LinkedIn, Facebook, Instagram), Pamphlet, Newsletter, Virtual information session, Report, Resource paper guide, In-person
Young Adult Cancer Canada (2001, 865086631 RR 0001)	Newfoundland & Labrador (Yes)	Patients, Caregivers, Siblings of patients, Healthcare Providers and English	Social media (Facebook, Instagram, Youtube, X), Videos, Blog, Newsletter, In-person, Virtual information sessions, Webinar, Training session
Zippaport (2023, 708664537 RR 0001)	Ontario (No)	Caregivers and English	Social media (Facebook, Instagram, Youtube), Newsletter

#### Step 4: Expert Consultations

**Figure 1.**
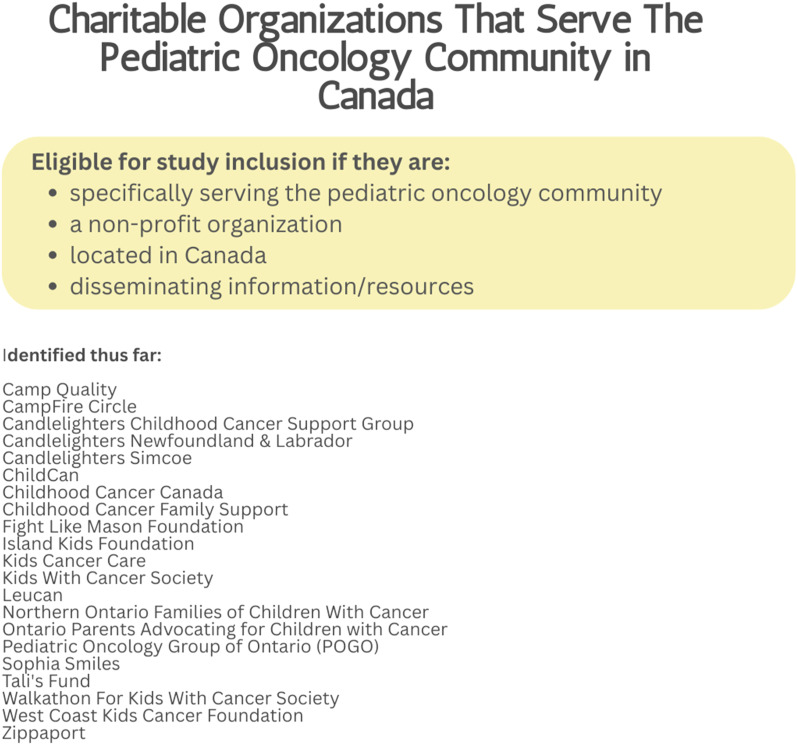
Poster created on Canva by C.F. and E.K.D. and posted on X, Instagram, Bluesky and LinkedIn for charitable organizations that serve the pediatric oncology community in Canada

Expert consultations were conducted to ensure the environmental scan fully captured the non-profit landscape in pediatric oncology in Canada and their knowledge dissemination activities.^27^ Members of the ACCESS network^22^ were asked via direct contact, newsletters and presentations to submit suggestions for consideration and to review our preliminary list of non-profits. Throughout the conduct of these activities, the list was constantly reviewed and updated. One of the co-authors (E.K.D.) is a co-founder of a global community for the adolescent and young adult cancer population (#AYACSM).^38^ Thus, using the author’s (E.K.D.) LinkedIn,^39^ Bluesky,^40^ Instagram,^41^ and X^42^ platforms, community members were asked to review and give suggestions to the list of organizations we identified through our search strategy (see Figure 1).^27^ The suggestions were screened and assessed for eligibility. In addition to this collaboration with experts in the community, our research team included members of pediatric oncology non-profit organizations and persons with lived experiences who helped review the organizations.

#### Step 5: Data Presentation and Visualization

The search strategy results were displayed using the Preferred Reporting Items for Systematic Reviews and Meta-Analyses extension for Scoping Reviews (PRISMA-ScR)^43^ flow diagram. The organizations’ characteristics were tabulated to demonstrate how many organizations were founded each decade, as well as their main audience for dissemination activities and the language(s) of the content and information they share. The data were also mapped to visualize the founding year of each specific foundation. Their modes of dissemination and the social media platforms used for the dissemination of activities are presented in graphical form.

### Ethical Considerations

Ethical approval was not required for this environmental scan, as it involved performing a grey-literature search to identify Canadian non-profit organizations and their various means of disseminating knowledge to the pediatric oncology community in Canada.^27^

### Patient and Public Involvement

Two of our study’s co-authors are non-profit partners from Leucan (P.C; Québec) and Kids Cancer Care (J.F; Alberta). Four of our study’s co-authors (C.F., S.R., K.E., and K.H.) are people with lived experiences of pediatric cancer who were reimbursed for their time. These experiences vary from being diagnosed with a pediatric cancer, providing care for a child with pediatric cancer, or providing care for a friend with pediatric cancer. Other authors are involved in the care, directed services, and/or conduct research in pediatric oncology. E.K.D. is the co-founder of the global adolescent and young adult cancer community, #AYACSM (adolescent and young adult cancer societal movement).^38^ All authors helped develop the study protocol^27^ and provided feedback on the final version of the manuscript.

## Results

**Figure 2.**
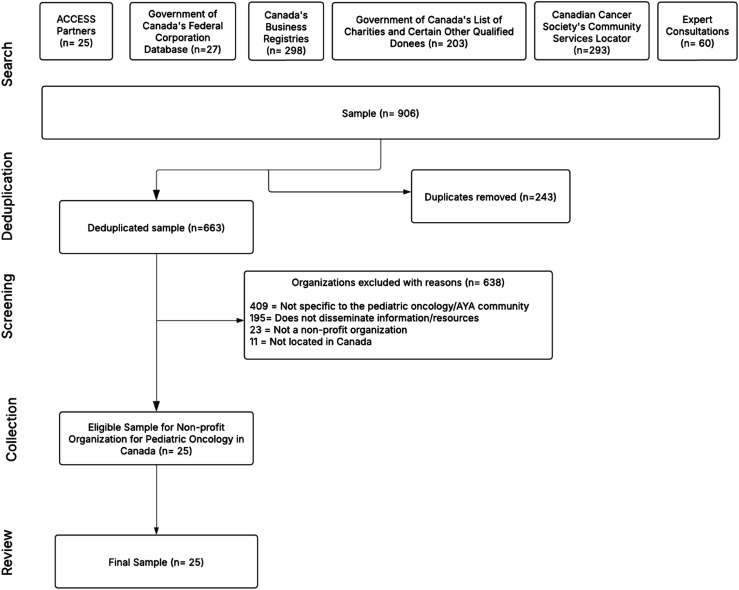
PRISMA-ScR Flow diagram of search and data collection strategy, adapted from Moher et al^36^

Our search strategy identified 906 results. Of these, 243 were duplicates that were removed and 638 were deemed ineligible as they did not meet our inclusion criteria. Of these, 409 results were not specific to the pediatric or AYA oncology community, 195 did not disseminate information and/or resources, 23 were not non-profit organizations, and 11 were not located in Canada (See Figure 2). Twenty-five organizations met our study’s inclusion criteria for data collection (See Table 2).^43^

### Non-Profit Organizational Characteristics

The headquarters of organizations identified in the environmental scan were primarily concentrated in Ontario (n = 11, 44%), followed by Québec (n = 4, 16%) and British Columbia (n = 3, 12.0%), as shown in Table 3. A smaller proportion of organizations’ headquarters were found in Alberta (n = 2, 8%), Manitoba (n = 2, 8%), Newfoundland & Labrador (n = 2, 8%), and Nova Scotia (n = 1, 4%). Provinces with organizations that have offices for subsidiary branches included Alberta (n = 1, 33%), Manitoba (n = 1, 33%) and Northwest Territories (n = 33%). Some regions showed no representation such as, New Brunswick, Prince Edward Island, Saskatchewan, Nunavut and Yukon.

**Table 3.** Characteristics of Pediatric Oncology Non-profit Organizations in Canada

Non-profit characteristic	Results
**Province/Territory of Headquarters**	
Ontario	11 (44%)
Québec	4 (16%)
British Columbia	3 (12%)
Alberta	2 (8%)
Manitoba	2 (8%)
Newfoundland & Labrador	2 (8%)
Nova Scotia	1 (4%)
New Brunswick	0
Prince Edward Island	0
Saskatchewan	0
Northwest Territories	0
Nunavut	0
Yukon	0
**Province/Territory of Organizations with Offices for Subsidiary Branches**	
Alberta	1 (33%)
Manitoba	1 (33%)
Northwest Territories	1 (33%)
**Year founded**	
1970-1979	2 (8%)
1980-1989	3 (12%)
1990-1999	4 (16%)
2000-2009	9 (36%)
2010-2019	5 (20%)
2020- Current (2025)	2 (8%)
**Language of content**	
English	18 (72%)
Both	7 (28%)
French	0
**Audience for content**	
Caregivers	25 (100%)
Patients	23 (92%)
Siblings of childhood cancer patients	18 (72%)
Healthcare providers	10 (40%)
Scientists	9 (36%)
Teachers	5 (20%)
Decision makers	2 (8%)
Indigenous community members	1 (4%)
**Organizations with Physical Office(s)**	
Yes	22 (88%)
No	3 (12%)
No PO Box	1
PO Box Only	2

**Figure 3.**
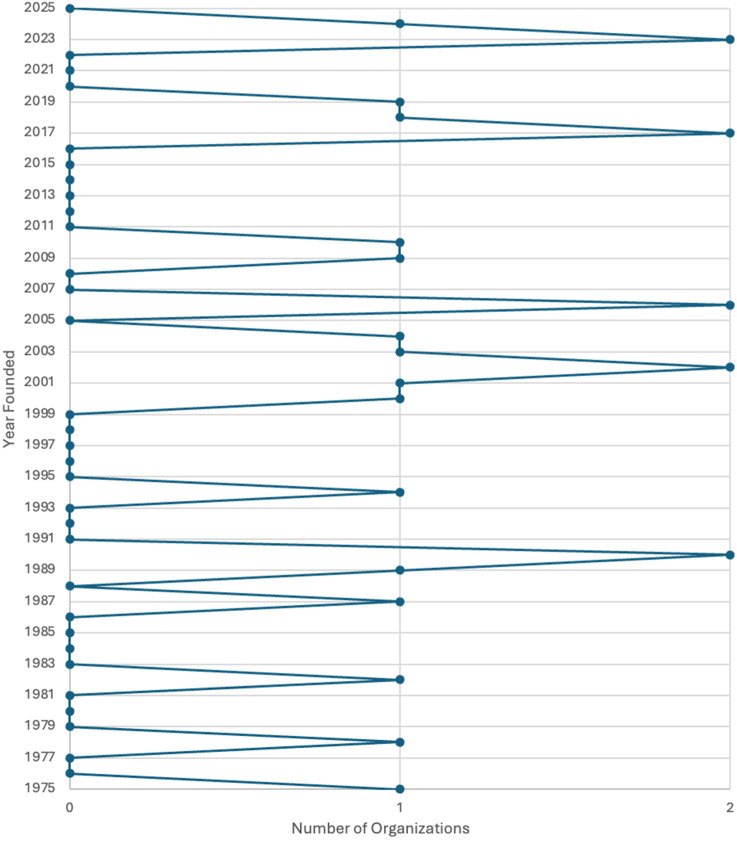
Organizations founded per year from 1975 to 2025

Most organizations were founded between 2000 and 2009, as shown in Figure 3 (n = 9, 36%). Regarding the language of dissemination, 72% of the organizations (n = 18) provided content solely in English, while only 28% (n = 7) offered bilingual resources (English and French). No other languages were used for dissemination activities. The audiences for these resources were predominantly caregivers (n=25, 100%), patients (n=23, 92%), and siblings of childhood cancer patients (n=18, 72%). Other audiences were healthcare providers (n=10, 40%), scientists (n=9, 36%), teachers (n=5, 20%), and decision makers (n=2, 8%). One organization had resources for Indigenous communities (n=1, 4%).

**Figure 4.**
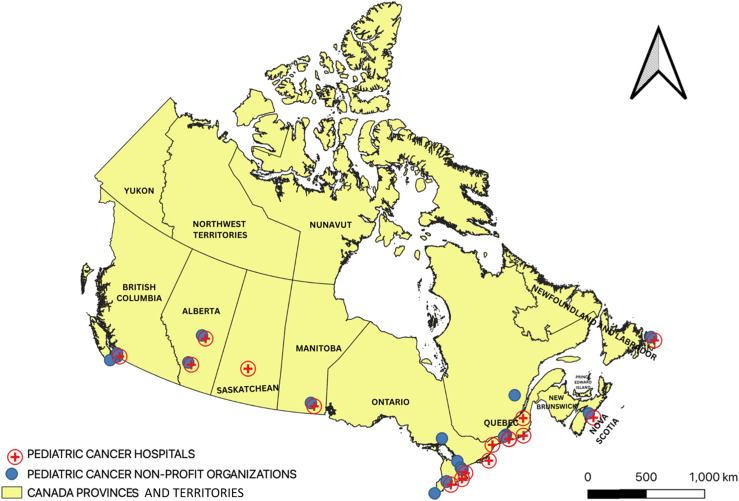
Map indicating the location of each non-profit organization and pediatric cancer centre in Canada for the pediatric oncology community

The map in Figure 4 showed the spatial distribution of organizations in Canada as well as the 16 pediatric cancer centres in the country. Seven provinces had a pediatric cancer centre and one or more non-profit organization in their region. In contrast, Saskatchewan was the only province that had a pediatric cancer centre, but no non-profit organization. Additionally, there were provinces and territories without pediatric centres that also lacked a pediatric cancer non-profit organization in their region.

### Knowledge Dissemination Strategies

**Figure 5.**
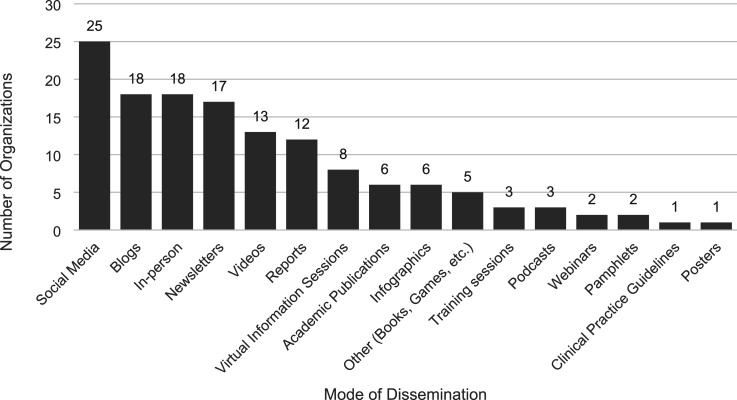
Modes of information dissemination utilized by non-profit organizations serving the childhood cancer community in Canada

**Figure 6.**
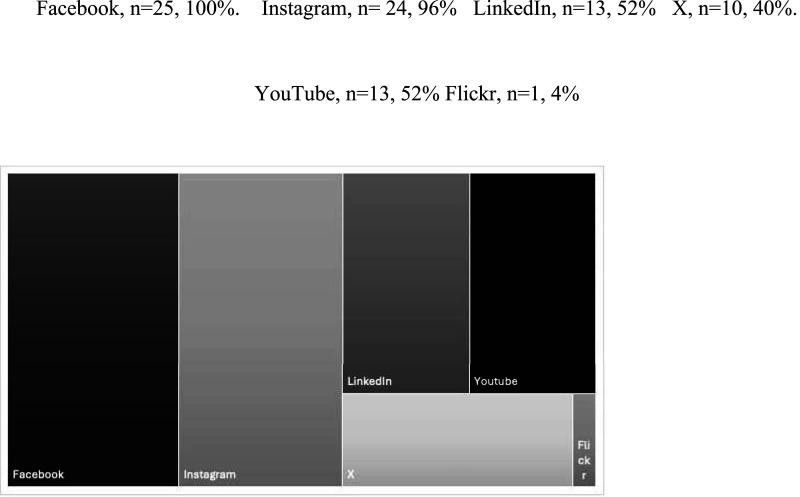
Percentage of social media platform usage for disseminating information by non-profit organizations

**Figure 7.**
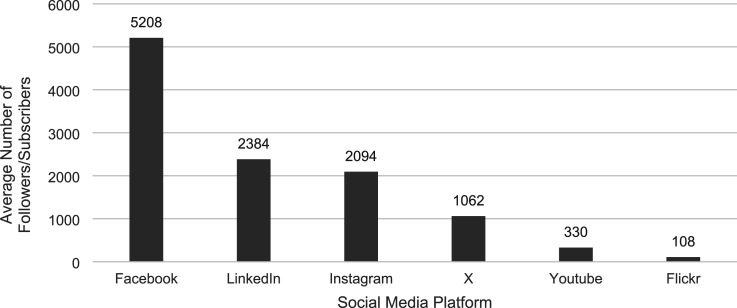
Average number of followers of each social media platform used by non-profit organizations in pediatric oncology for knowledge dissemination. *Facebook, LinkedIn, Instagram and X were assessed with followers. YouTube was assessed with subscribers. Retrieved June-July 2025

**Figure 8.**
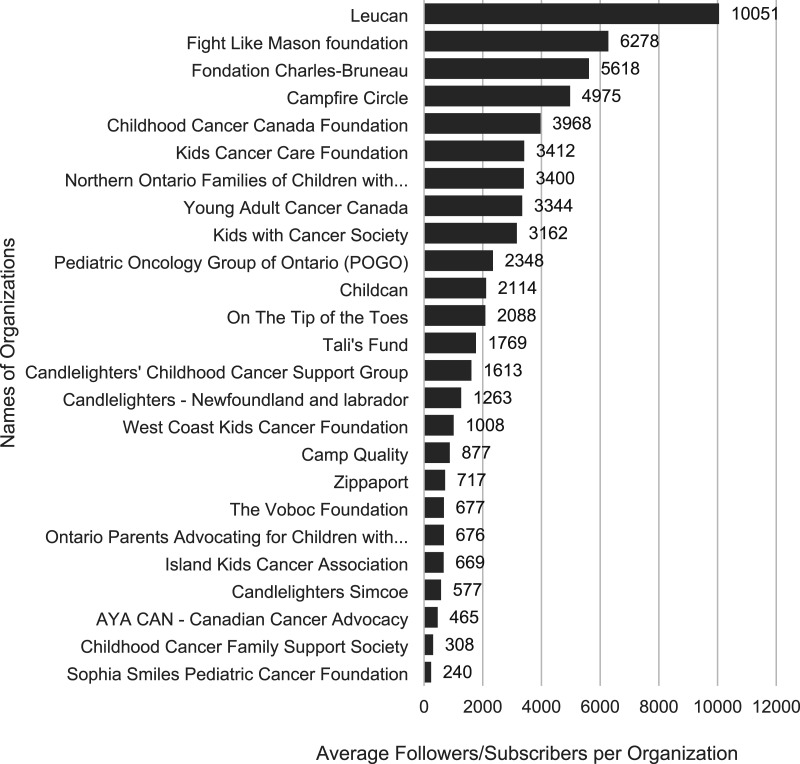
Average number of social media followers across platforms per non-profit organizations in pediatric oncology for dissemination

The knowledge dissemination strategies used by the 25 non-profit organizations were predominantly digital and in-person. As illustrated in Figure 5, all organizations utilized some form of social media (n = 25,100%) for dissemination activities.^44-68^ Furthermore, a significant portion of these organizations used blogs (n = 18, 72%),^44,46,47,49-52,54,55,57-64,68^ in-person activities (n=18, 72%),^44,46-50,54,55,57-62,64-66,68^ newsletters (n=17, 68%),^44-47,49,50,52,55,57,59,60,62,64-68^ videos (n=13, 52%)^44,47,49,51-57,62,64,65^ and their websites (n=25; 100%)^44-68^ as communication channels. Organizations also used reports (n=12, 48%),^46-48,51,52,55,58,59,61,62,66,68^ virtual information sessions (n=8, 32%),^44-47,56,61,64,66^ infographics (n=6, 24%),^46,52,54,57,61,68^ academic publications (n=6, 24%),^45,51,57,62,64,68^ podcasts (n=3, 12%),^45,54,56^ training sessions (n=3, 12%),^44,45,49^ pamphlets (n=2, 8%),^46,66^ webinars (n=2, 8%),^44,58^ posters (n=1, 4%),^46^ clinical practice guidelines (n=1, 4%),^62^ and other methods (n=5, 20%)^45,49,52,59,66^ such as books, games, etc. However, policy briefs were not used by organizations.^27^ Additionally, as indicated in Figure 6, social media platforms, particularly Facebook^69^ (n = 25, 100 %)^44-68^ and Instagram^41^ (n = 24, 96%),^44-59,61-68^ were the most frequently used methods for engaging with the community. Other platforms used by organizations were LinkedIn^39^ (n=13, 52%),^45-47,51,52,55,57,59,62,64-66,68^ YouTube^70^ (n=13, 52%)^44,46,47,54,55,57,59,61,62,64,65,67,68^ and X^42^ (n=10, 40%),^44-49,54,55,61,62^ previously known as Twitter, and Flickr^71^ (n=1, 4%).^68^ Snapchat,^72^ Threads,^73^ Bluesky^40^ and TikTok^74^ were not used.^27^ Social media followings (described as followers or subscribers depending on the social media platform) were highest on Facebook,^69^ with an average following of 5,208 across all organizations, as indicated in Figure 7. The platform with the second highest follower count was LinkedIn^39^ with an average of 2384. The average following for Instagram,^41^ X,^42^ YouTube^70^ and Flickr^71^ were 2094 followers, 1062 followers, 330 subscribers, and 108 followers, respectively. Figure 8 demonstrates the average number of social media followers across all platforms for each non-profit organization.

## Discussion

This environmental scan revealed significant insights into the landscape of 25 non-profit organizations serving Canada’s pediatric oncology community and their knowledge dissemination strategies. Despite the significant benefits and strengths of non-profits’ dissemination efforts in pediatric cancer such as providing patients and communities with in-person and online resources locally, many gaps persist in their knowledge dissemination activities. One notable limitation is the underrepresentation of organizations that offer bilingual content, which can create inequities in access for francophone communities, There are over eight million Canadians reporting French as their first official language in 2021.^75^ While many reside in Quebec, Francophone communities are present in all provinces and territories across Canada.^75^ Existing non-profit organizations primarily provide information in English, with 18 out of 25 organizations offering content only in this official language. In comparison, a smaller subset of seven organizations provides resources in both official languages of English and French. Québec, a French-speaking province, accounts for approximately 20% of the Canadian population. Moreover, just over 6.5 million individuals nationwide reported being bilingual (English and French),^75^ which results in approximately 1.5 million Canadians who may be unable to benefit from the information shared by most of the non-profit organizations that provide content in English only. According to the government of Canada, non-profit organizations’ efforts may be in the official language of their choice.^76^ No non-profit organizations offered resources in Indigenous languages or other non-official languages (e.g., Korean, Persian, Mandarin) important to multi-cultural, multi-lingual Canadian population.^77,78^ Advancements in large language models may offer multilingual support, but ongoing work is needed to provide safe, accurate, reliable, readable, and culturally relevant health information.^79,80^ Presently, the information still needs to be verified with health care providers but opportunities remain to improve the accessibility and quality of health care information within pediatric oncology care using large language models.^81^

There is an uneven geographic distribution of non-profit organizations, with only seven of the 13 provinces/territories having a head office of an organization based in their province and most non-profits located in Ontario. The Northwest Territories, Alberta and Manitoba are the only provinces that have offices for subsidiary branches. Therefore, provinces like New-Brunswick and Saskatchewan have limited access to in-person resources. The disparities in regional access to non-profit organizations may lead to resource inequalities. Despite all organizations being online, geographical representation is important as some information is disseminated in-person, in hospitals or offices. Therefore, identifying underserved areas may prompt existing non-profits to expand their mandate and inform the future development of non-profit organizations provincially or in regional settings (e.g. rural). Local cultures may not be appropriately represented in nationwide organizations. Using New Brunswick as an example, in 2021, the province had over 33,000 Indigenous people, with the Mi’kmaq, Wolastoqey (Maliseet), and Passamaquoddy being the three main Indigenous groups.^82,83^ The only resources for Indigenous communities were created by an Ontario organization.^62^ Thus, as the province has no resources available for this community, they may have been poorly represented by organizations in the region. More generally, Statistics Canada mentions that three-quarters (75.1%) of non-profit organizations in rural and small towns in Canada are in five provinces: Québec (24.4%), Ontario (19.5%), Alberta (12.1%), Saskatchewan (9.6%) and British Columbia (9.5%).^84^ These statistics highlight the lack of organizations in other provinces. Therefore, non-profit organizations that share information with diverse regional cultures are crucial in supporting everyone within the pediatric oncology community.^21^

Most non-profit organizations actively employ the use of digital platforms to disseminate information, such as online blogs (n = 18, 72 %), and other social media platforms (e.g., Facebook^69^ (n = 25, 100%), and Instagram^41^ (n = 24, 96%)). The use of online dissemination strategies is important as families, particularly those with lower health literacy, often turn to the Internet and social media for information.^19^ By providing digital resources, these organizations enhance accessibility and reach, aligning with evidence indicating that 98% of family caregivers use internet-based information to search for cancer-related information when their child is initially diagnosed.^18^ While the format and accessibility of disseminated digital information may benefit some, they also create barriers for others. Digital platforms like social media and blogs are commonly used, but not accessible to everyone, particularly individuals between the ages of 50 to 64. As per Statistics Canada, 6 in 10 individuals between the age of 50 to 64 use social media^85^ and only 25% of individuals older than 55 years old reported having good vision without correction.^86^ Therefore, caregivers in that age range may miss important information being disseminated online by non-profit organizations. Thus, an organization may use different strategies to reach these audiences, such as webinars, in-person information sessions, podcasts, videos and others. These strategies may offer communication opportunities to ask any questions and help convey knowledge to the audience.^87^

As seen in Figure 3, there has been an increase in the formation of non-profit organizations in Canada over the past 50 years. However, according to the 2024 Giving Report, the number of Canadians donating to charitable organizations has decreased.^88^ There has been a positive association between giving and social networks. When people have a social network of six or more, 80% of them donate to organizations.^88^ On the other hand, in 2023, one in five Canadians used charitable organizations to meet essential needs, and 57% of organizations were unable to meet their demands.^72^ Therefore, it is important for charities to grow and share their stories for Canadians to make donations to their cause, as well as create policies and initiatives to allow Canadians to foster deeper connections with their community. According to the Childhood Cancer Canada Annual Report for 2022,^89^ their aid for kids with cancer was nationwide, with almost 800 families receiving financial assistance via this organization, demonstrating the impact and importance of non-profit organizations on communities. Moreover, despite the decrease in donations, there has been a shift to online giving since the pre-pandemic period, creating a new way for non-profit organizations to receive help from their communities.^88^ Social media may also play an important role in gathering donations. The use of Facebook,^69^ Instagram^41^ and other online platforms, which were used by the identified organizations in this environmental scan, can help raise money that is vital for sustainability.

### Strengths and Limitations

The active involvement of community members and persons with lived experiences in developing and refining our methodology and analysis underscores a positive shift toward more participatory research practices.^88^ Opportunities to further strengthen this work could have included the adoption of a checklist for patient and public involvement in research. Our post hoc evaluation using the GRIPP2,^90^ suggested areas of improvement could have been reporting the specific positive and negative results of the involvement of patients and members of the public if any contextual factors enabled or hindered their impact in our study. This strengthens the relevance and impact of the findings and encourages collaboration in healthcare advocacy.^88^ Despite the valuable insights provided by this environmental scan, notable limitations warrant consideration. Firstly, while our search strategy was comprehensive, we may have missed organizations that were eligible for inclusion. Additionally, some organizations were deemed ineligible for inclusion as they did not have a registered charitable number, but they may have a significant impact on knowledge mobilization, disseminating resources and content, as well as advocacy initiatives. Therefore, it is possible that we failed to capture all dissemination efforts. Also, this study was not designed to thoroughly examine the quality and content of the resources disseminated by the included non-profit organizations, which may be of interest for further research. Opportunities to assess the quality and credibility of the content can be done with tools such as DISCERN,^91^ HONcode,^92^ and PEMAT.^93^ For example, Hargrave et al assessed 114 websites (35% charitable) about childhood brain tumours using DISCERN and found the topics were deficient in covering etiology, late effects, prognosis, and treatment choices.^94^ Understanding the substance and reliability of these materials is crucial for evaluating their potential impact. Additionally, we did not explore the effectiveness of the dissemination strategies employed by these organizations, which means we lack insight into how well these strategies succeeded in reaching their intended audience. This gap in the evaluation of the dissemination strategies limits our ability to fully understand the overall effectiveness and reach of the initiatives in question.

Implications for knowledge dissemination of research, particularly within pediatric oncology, underscore the need for a more unified approach. We need to take advantage of the existing channels of communication, such as social media, blogs, and newsletters, which are essential avenues for storytelling, sharing research findings and advocacy efforts. However, it is equally important to explore creative and new platforms that can broaden the reach of these non-profit organizations to reach different audiences and leverage the expertise of these organizations who serve as trustworthy knowledge brokers for the childhood cancer community. A central hub with a list of all the organizations and links to their website may improve equitable dissemination of the latest knowledge in the pediatric oncology community. This centralized pan-Canadian platform could help ensure that all, regardless of geographical location and preferred official language, receive the latest evidence in plain (lay terms) and scientific language.^95^ Future opportunities include amplifying the accessibility of the knowledge being disseminated using large language models for global sharing and exchange.^79^

## Conclusion

In conclusion, this environmental scan has provided valuable insights into the landscape of non-profit organizations dedicated to pediatric oncology in Canada and their knowledge dissemination efforts. By employing a methodology that combines both Internet-based searches and expert consultations, we identified 25 active non-profit organizations with headquarters across seven provinces, largely serving English-speaking communities. Our findings emphasize the strengths of current knowledge dissemination efforts, especially the extensive use of digital platforms for disseminating information, which enhances accessibility for families affected by pediatric cancer. However, several gaps have been identified, such as the limited availability of French resources and uneven geographic distribution. These gaps underscore the need for ongoing improvements in the equitable distribution of information and resources to support families throughout Canada. Looking ahead, addressing these gaps by expanding bilingual content, improving regional representation, and developing a centralized directory of organizations will be crucial for strengthening overall knowledge mobilization efforts in pediatric oncology and ensuring all families have access to the information and support they require.

## Supplemental Material

Supplemental Material - Pediatric Oncology Knowledge Mobilization in Canada: A Environmental Scan Supplemental Material for Pediatric Oncology Knowledge Mobilization in Canada: A Environmental Scan by Catherine Foulem, Emily K. Drake, Angelina Lui, Sharon Dias, Ekaterini Damoulianos, Stephanie Reid, Patrick Cossette, Michel Duval, Kirsten Efremov, James Foster, Karen Haas, Argerie Tsimicalis on behalf of the ACCESS (Advancing Childhood Cancer Experience, Science & Survivorship) Network in The Journal of Health Care Organization, Provision, and Financing.

Supplemental Material - Pediatric Oncology Knowledge Mobilization in Canada: A Environmental Scan Supplemental Material for Pediatric Oncology Knowledge Mobilization in Canada: A Environmental Scan by Catherine Foulem, Emily K. Drake, Angelina Lui, Sharon Dias, Ekaterini Damoulianos, Stephanie Reid, Patrick Cossette, Michel Duval, Kirsten Efremov, James Foster, Karen Haas, Argerie Tsimicalis on behalf of the ACCESS (Advancing Childhood Cancer Experience, Science & Survivorship) Network in The Journal of Health Care Organization, Provision, and Financing.

## Data Availability

All data from this environmental scan have been made available through this manuscript via tables, figures, a description of the results and the supplementary files.*

## Appendix

### Abbreviations

KMb =: Knowledge mobilization

## References

[1] Ellison L Xie L Sung L . Trends in paediatric cancer survival in Canada, 1992 to 2017. Catalogue no. 82-003-X. Health Reports. Statistics Canada. 17 . February ; 2021. doi:10.25318/82003x202100200001eng accessed 9 October 2024. 33595224

[2] Klein-Geltink J Shaw AK Morrison HI Barr RD Greenberg ML . Use of paediatric versus adult oncology treatment centres by adolescents 15–19 years old: the Canadian Childhood Cancer Surveillance and Control Program. European Journal of Cancer. 2005;41:404-410. 15691640 10.1016/j.ejca.2004.10.023

[3] Public Health Agency of Canada . Childhood Cancer Counts in Canada. Infographic, Government of Canada. https://www.canada.ca/en/public-health/services/publications/diseases-conditions/childhood-cancer-counts-canada.html. 14 February 2022. accessed 5 December 2024.

[4] Ritter J Allen S Cohen PD , et al. Financial hardship in families of children or adolescents with cancer: a systematic literature review. The Lancet Oncology. 2023;24:e364-e375. 37657477 10.1016/S1470-2045(23)00320-0 PMC10775706

[5] Lewandowska A . Influence of a Child’s Cancer on the Functioning of Their Family. Children (Basel). 2021;8:592. 34356571 10.3390/children8070592 PMC8306515

[6] Loeb S Langford AT Bragg MA Sherman R Chan JM . Cancer misinformation on social media. CA A Cancer J Clinicians. 2024;74:453-464. 10.3322/caac.21857 PMC11648589 38896503

[7] Teplinsky E Ponce SB Drake EK , et al. Online Medical Misinformation in Cancer: Distinguishing Fact From Fiction. JCO Oncology Practice. 2022;18:584-589. 35357887 10.1200/OP.21.00764 PMC9377685

[8] Boeriu E Borda A Miclea E , et al. Prognosis Communication in Pediatric Oncology: A Systematic Review. Children (Basel). 2023;10:972. 37371204 10.3390/children10060972 PMC10297328

[9] Bell J Condren M . Communication Strategies for Empowering and Protecting Children. J Pediatr Pharmacol Ther. 2016;21:176-184. 27199626 10.5863/1551-6776-21.2.176 PMC4869776

[10] Carter B Young S Ford K Campbell S . The Concept of Child-Centred Care in Healthcare: A Scoping Review. Pediatric Reports. 2024;16:114-134. 38391000 10.3390/pediatric16010012 PMC10885088

[11] Loeffen Ea. H Kremer LCM Mulder RL , et al. The importance of evidence-based supportive care practice guidelines in childhood cancer-a plea for their development and implementation. Support Care Cancer. 2017;25:1121-1125. 27928642 10.1007/s00520-016-3501-y PMC5321691

[12] Knowledge mobilisation framework. 2022. https://preventioncentre.org.au/. https://preventioncentre.org.au/resources/knowledge-mobilisation-framework/. accessed 15 April 2025.

[13] Ziam S Lanoue S McSween-Cadieux E , et al. A scoping review of theories, models and frameworks used or proposed to evaluate knowledge mobilization strategies. Health Res Policy Sys 22: doi:10.1186/s12961-023-01090-7. (Epub ahead of print 10 January 2024). PMC10777658 38200612

[14] Golhasany H Harvey B . Capacity development for knowledge mobilization: a scoping review of the concepts and practices. Humanit Soc Sci Commun. 2023;10:1-12.

[15] Cooper A Rodway J Read R . Knowledge Mobilization Practices of Educational Researchers Across Canada. Canadian journal of higher education. 2018;48:1-21.

[16] Gagnon M . Knowledge dissemination and exchange of knowledge. 2010. https://www.cihr-irsc.gc.ca/e/41953.html

[17] Marín-González E Malmusi D Camprubí L Borrell C . The Role of Dissemination as a Fundamental Part of a Research Project: Lessons Learned From SOPHIE. Int J Health Serv. 2017;47:258-276. 27799595 10.1177/0020731416676227

[18] Rodgers CC Laing CM Herring RA , et al. Understanding Effective Delivery of Patient and Family Education in Pediatric OncologyA Systematic Review From the Children’s Oncology Group [Formula: see text]. J Pediatr Oncol Nurs. 2016;33:432-446. 27450361 10.1177/1043454216659449 PMC5235950

[19] Chen X Hay JL Waters EA , et al. Health Literacy and Use and Trust in Health Information. J Health Commun. 2018;23:724-734. 30160641 10.1080/10810730.2018.1511658 PMC6295319

[20] Nordin N Khatibi A Azam SMF . Nonprofit capacity and social performance: mapping the field and future directions. Manag Rev Q. 2024;74:171-225.

[21] Ressler RW Paxton P Velasco K Pivnick L Weiss I Eichstaedt JC . Nonprofits: A Public Policy Tool for the Promotion of Community Subjective Well-being. J Public Adm Res Theory. 2021;31:822-838. 34608375 10.1093/jopart/muab010 PMC8482971

[22] ACCESS . Advancing Childhood Cancer Experience, Science & Survivorship. https://www.accessforkidscancer.ca/

[23] Peters MDJ Marnie C Tricco AC , et al. Updated methodological guidance for the conduct of scoping reviews. JBI Evid Synth. 2020;18:2119-2126. 33038124 10.11124/JBIES-20-00167

[24] Castro A Lalonde-LeBlond G Freitas Z , et al. In-Home Respite Care Services Available to Families With Palliative Care Needs in Quebec: Novel Digital Environmental Scan. JMIR Nurs. 2024;7:e53078. 38625735 10.2196/53078 PMC11061788

[25] Choo C . Environmental scanning as information seeking and organizational learning. Information Research. 2001;7. accessed 6 January 2025.

[26] Rowel R Moore ND Nowrojee S Memiah P Bronner Y . The utility of the environmental scan for public health practice: lessons from an urban program to increase cancer screening. J Natl Med Assoc. 2005;97:527-534. 15868772 PMC2568702

[27] Drake E Foulem C Damoulianos E , et al. Pediatric Oncology Knowledge Mobilization in Canada: An Environmental Scan Protocol (Preprint). Epub ahead of print 30 April 2025. doi:10.2196/preprints.76787.

[28] Charlton P Doucet S Azar R , et al. The use of the environmental scan in health services delivery research: a scoping review protocol. BMJ Open. 2019;9:e029805. 10.1136/bmjopen-2019-029805 PMC6731933 31494613

[29] von EE Altman DG Egger M Pocock SJ Gøtzsche PC Vandenbroucke JP . The Strengthening the Reporting of Observational Studies in Epidemiology (STROBE) statement: guidelines for reporting observational studies. J Clin Epidemiol. 2008;61:344-349. 18313558 10.1016/j.jclinepi.2007.11.008

[30] Agency CR . What is the difference between a registered charity and a non-profit organization? 2016. https://www.canada.ca/en/revenue-agency/services/charities-giving/about-registered-charities/what-difference-between-a-registered-charity-a-non-profit-organization.html. accessed 5 February 2025.

[31] What Should the Age Range Be for AYA Oncology? Journal of Adolescent and Young Adult Oncology 2011; 1: 3–10. 26812562 10.1089/jayao.2011.1505

[32] Our Partners. In: Access. https://www.accessforkidscancer.ca/about-us/our-partners/. accessed 6 January 2025.

[33] Government of Canada CI of HR . Welcome to the Canadian Institutes of Health Research - CIHR. 2003. https://cihr-irsc.gc.ca/e/193.html. accessed 6 January 2025.

[34] Search for a Federal Corporation - Online Filing Centre - Corporations Canada - Corporations - Innovation, Science and Economic Development Canada. https://ised-isde.canada.ca/cc/lgcy/fdrlCrpSrch.html accessed 8 January 2025.

[35] Canada’s Business Registries. https://canadasbusinessregistries.ca/search accessed 6 January 2025.

[36] Agency CR . List of charities and certain other qualified donees - basic search. https://apps.cra-arc.gc.ca/ebci/hacc/srch/pub/dsplyBscSrch?request_locale=en. accessed 5 February 2025.

[37] Canadian Cancer Society . Community Service Locator. https://csl.cancer.ca/en

[38] Drake EK Weeks LE van Manen M Taylor D Ricci I Curran J . How Advocates Can Support Young Adults Living With Cancer and Their Transition to Palliative Care. Qual Health Res. 2025;35:1007-1018. 39499809 10.1177/10497323241279083 PMC12202822

[39] LinkedIn. https://www.linkedin.com/home

[40] Bluesky. https://bsky.app/

[41] Instagram. https://www.instagram.com/

[42] X. https://x.com/

[43] Tricco AC Lillie E Zarin W , et al. PRISMA Extension for Scoping Reviews (PRISMA-ScR): Checklist and Explanation. Ann Intern Med. 2018;169:467-473. 30178033 10.7326/M18-0850

[44] Young Adult Cancer Canada. https://youngadultcancer.ca/

[45] AYA CAN - Canadian cancer advocacy. https://ayacan.ca/

[46] Camp Quality. https://www.campquality.org/

[47] Campfire Circle. https://campfirecircle.org/about-campfire-circle/

[48] Candlelighters - Newfoundland and labrador. https://www.candlelightersnl.ca/

[49] Candlelighters Simcoe. https://www.candlelighterssimcoe.ca/

[50] Candlelighters’ childhood cancer support group. https://www.manitobacandlelighters.org/

[51] Childcan. https://childcan.com/

[52] Childhood Cancer Canada Foundation - Fondation canadienne du Cancer Chez l’enfant. https://www.childhoodcancer.ca/

[53] Childhood cancer family support society. https://ccfsupport.com/

[54] Fight like Mason foundation. https://fightlikemason.org/

[55] Fondation Charles-Bruenau. https://www.charlesbruneau.qc.ca/fr/

[56] Island Kids Cancer Association. https://ikca.ca/programs/

[57] Kids Cancer Care Foundation. https://www.kidscancercare.ab.ca/?gad_source=1&gclid=CjwKCAiAtsa9BhAKEiwAUZAszfB9Mrqfb9FsiJrdSP38NR4igekDnSTs5HCoQGeu-BCGxU3X_naXQRoCidIQAvD_BwE

[58] Kids with cancer society. https://www.kidswithcancer.ca/

[59] Leucan. https://www.leucan.qc.ca/en/

[60] Northern Ontario families of children with cancer (NOFCC). https://nofcc.ca/

[61] Ontario Parents Advocating for Children with Cancer (OPACC). https://www.opacc.org/

[62] Pediatric Oncology Group of Ontario (POGO). https://www.pogo.ca/

[63] Sophia Smiles Pediatric Cancer Foundation. https://www.sophiasmiles.org/

[64] Tali’s Fund. https://talisfund.org/about-us/what-we-do/

[65] Voboc Foundation. https://voboc.org/

[66] West Coast Kids Cancer Foundation. https://wckfoundation.ca/

[67] Zippaport - port shorts for brave kids. https://www.zippaport.ca/

[68] On The Tip Of The Toes. https://tipoftoes.com/

[69] Facebook. https://www.facebook.com/

[70] Youtube. https://www.youtube.com/

[71] Flickr. https://www.flickr.com/

[72] Snapchat. https://www.snapchat.com/

[73] Threads. https://www.threads.com/?hl=en

[74] TikTok. https://www.tiktok.com/en/

[75] Government of Canada . Statistics on official languages in Canada. 2024. https://www.canada.ca/en/canadian-heritage/services/official-languages-bilingualism/publications/statistics.html

[76] Government of Canada . Creating a not-for-profit corporation. 2022-07-21.

[77] Statistics Canada Hou F Schimmele C Stick M . Changing demographics of racialized people in Canada. Epub ahead of print 2023. August 23, 2023 doi:10.25318/36280001202300800001-ENG

[78] Statistics Canada . Non-official languages spoken at home in Canada, 2021.

[79] Lawson MLA Yen TL . Machine Translation for Multilingual Cancer Patient Education: Bridging Languages, Navigating Challenges. J Canc Educ. 2024;39:477-478. 10.1007/s13187-024-02438-5 PMC11461557 38652432

[80] Menz BD Modi ND Abuhelwa AY , et al. Generative AI chatbots for reliable cancer information: Evaluating web-search, multilingual, and reference capabilities of emerging large language models. European Journal of Cancer. 2025;218:115274. 39922126 10.1016/j.ejca.2025.115274

[81] Sezgin E Jackson DI Kocaballi AB , et al. Can Large Language Models Aid Caregivers of Pediatric Cancer Patients in Information Seeking? A Cross-Sectional Investigation. Cancer Med. 2025;14:e70554. 39776222 10.1002/cam4.70554 PMC11705392

[82] Statistics Canada . Focus on Geography Series, 2021 Census of Population, New-Brunswick, Province. 2022. https://www12.statcan.gc.ca/census-recensement/2021/as-sa/fogs-spg/page.cfm?lang=E&topic=8&dguid=2021A000213

[83] Government of New Brunswick . First Nations communities. https://www.gnb.ca/en/topic/culture-heritage/indigenous-peoples/first-nations-communities.html#:∼:text=Learn_more_about_the_First,Peskotomuhkati_Nation_at_Skutik

[84] Statistics Canada . Non-profit organizations in rural and small town Canada. 2022. https://www150.statcan.gc.ca/n1/daily-quotidien/250217/dq250217c-eng.htm

[85] Schimmele F . Schellenberg. Canadians’ assessments of social media in their lives. Statistics Canada. 2021. https://www150.statcan.gc.ca/n1/pub/36-28-0001/2021003/article/00004-eng.htm

[86] Statistics Canada Finès P . Self-reported eye health in Canada: 20 years of data. Epub ahead of print 2022. April 20, 2022. doi:10.25318/82-003-X202200400002-ENG 35442610

[87] Sivaramalingam J Rajendiran KS Mohan M , et al. Effect of webinars in teaching-learning process in medical and allied health science students during COVID-19 pandemic: A cross-sectional study. J Educ Health Promot. 2022;11:274. 36325216 10.4103/jehp.jehp_1450_21 PMC9621355

[88] CanadaHelps . The 2024 Giving Report. 2024. https://indd.adobe.com/view/763060b8-d8a0-48c9-8325-c9e619340e9a

[89] Childhood Cancer Canada . Annual Report 2022. 2022. https://www.childhoodcancer.ca/wp-content/uploads/2024/06/CCC-AnnualReport-2022.pdf

[90] Staniszewska S Brett J Simera I , et al. GRIPP2 reporting checklists: tools to improve reporting of patient and public involvement in research. BMJ. 2017;358:j3453. 28768629 10.1136/bmj.j3453 PMC5539518

[91] Shepperd S Charnock D . Why DISCERN? Health Expect. 1998;1:134-135. 11281867 10.1046/j.1369-6513.1998.0112a.x PMC5139898

[92] Boyer C Selby M Scherrer J-R Appel RD . The Health On the Net Code of Conduct for medical and health Websites. Computers in Biology and Medicine. 1998;28:603-610. 9861515 10.1016/s0010-4825(98)00037-7

[93] Shoemaker SJ Wolf MS Brach C . The Patient Education Materials Assessment Tool (PEMAT) and User’s Guide. October 2013. 10.1016/j.pec.2014.05.027 PMC5085258 24973195

[94] Hargrave DR Hargrave UA Bouffet E . Quality of health information on the Internet in pediatric neuro-oncology. Neuro Oncol. 2006;8:175-182. 16533758 10.1215/15228517-2005-008 PMC1871939

[95] Graetz DE Caceres‐Serrano A Radhakrishnan V Salaverria CE Kambugu JB Sisk BA . A proposed global framework for pediatric cancer communication research. Cancer. 2022;128:1888-1893. 35201609 10.1002/cncr.34160 PMC9303244

