# Supplementary material for: Pediatric Oncology Knowledge Mobilization in Canada: A Environmental Scan
Source: Inquiry. 2026 Jul 15;63:00469580261468769. doi: 10.1177/00469580261468769 (PMC13373428; doi:10.1177/00469580261468769)
Supplement: Supplemental Material - Pediatric Oncology Knowledge Mobilization in Canada: A Environmental Scan [file sj-pdf-1-inq-10.1177_00469580261468769.pdf]

Table 1: Social Media Followers and Names of Identified Non-Profit Organizations Serving the Pediatric Oncology Community

Organization	Facebook	Facebook followers	Instagram	Instagram Followers	LinkedIn	LinkedIn Followers	X (formerly Twitter)	X Followers	Youtube	Youtube Subscribers	Flickr	Followers
AYA CAN - Canadian Cancer Advocacy	https://www.facebook.com/ayacan.cancer/	124	https://www.instagram.com/ayacan_cancer/	1279	https://www.linkedin.com/company/aya-can-canadian-cancer-advocacy/	231	https://x.com/ayacan_cancer	227				
Camp Quality	https://www.facebook.com/CampQualityCanada/	2,300	https://www.instagram.com/cqcanada/?hl=en	918	https://www.linkedin.com/company/camp-quality-canada	832	https://x.com/CampQualityCan?ref_src=twsrc%5Egoogle%7Ctwcamp%5Eserp%7Ctwgr%5Eauthor	302	https://www.youtube.com/channel/UCvP-zYDD0tdmIgotysBCM4g	33		
Campfire Circle	https://www.facebook.com/campfirecircleorg/	9,600	https://www.instagram.com/ca	10, 600	https://www.linkedin.com/company/campfirecircleorg/	6,335	https://x.com/Campfirecircle_	3,373	https://www.youtube.com/c/Campfi	593		

			mpfirecircleorg/						reCircle TV			
Candlelighters - Newfoundland and Labrador	https://www.facebook.com/CandlelightersNL/	2,400	https://www.instagram.com/candlelightersnl/	840			https://x.com/CandlelighterNL	549				
Candlelighters Simcoe	https://www.facebook.com/CandlelightersSimcoe/	1,000	https://www.instagram.com/candlelighterssimcoe/	550			https://x.com/clighterssimcoe	182				
Candlelighters' Childhood Cancer Support Group	https://www.facebook.com/manitobacandlelighters	2,400	https://www.instagram.com/manitobacand	826								

			lelighters/#									
Childcan	https://www.facebook.com/Childcan/	4,200	https://www.instagram.com/childcan/	1,633	https://www.linkedin.com/in/childcan/?original_referer=https%3A%2F%2Fchildcan.com%2F	508						
Childhood Cancer Canada	https://www.facebook.com/ChildhoodCancerCanada	6,800	https://www.instagram.com/childhoodcancercanada/	3,103	https://www.linkedin.com/company/childhood-cancer-canada/	2,000						

Childhood Cancer Family Support Society	https://www.facebook.com/childhoodcancerfamilysupport/	629	https://www.instagram.com/childhoodcancerfamilysupport/	203			https://x.com/CCFSupport	92				
Fight Like Mason Foundation	https://www.facebook.com/TeamMaseMan/	17,000	https://www.instagram.com/fight.like.mason/	7,874			https://x.com/fightlikemason	226	https://www.youtube.com/channel/UCo31lqku4_aeoX3OL-PNlvQ	13		
Fondation Charles-Bruneau	https://www.facebook.com/fcharlesbruneau	17,000	https://www.instagram.com/fcharlesbruneau/	4,071	https://www.linkedin.com/company/fcharlesbruneau/	5,000	https://x.com/fcharlesbruneau	1,517	https://www.youtube.com/user/fcharlesbruneau	501		

Island Kids Cancer Association	https://www.facebook.com/IKCA2017	430	https://www.instagram.com/islandkidscancerassociation/	907								
Kids Cancer Care	https://www.facebook.com/KCCFA	6,400	https://www.instagram.com/kidscancercare/	3,765	https://www.linkedin.com/company/kids-cancer-care-foundation-of-alberta/	3,000			https://www.youtube.com/user/KidsCancerCare	482		
Kids with Cancer Society	https://www.facebook.com/kwcsyeg	3,900	https://www.instagram.com/kwcsyeg/	2,423								

Leucan	https://www.facebook.com/Leucanpageprovinciale/	23,000	https://www.instagram.com/leucan/	8,262	https://www.linkedin.com/company/leucan/	8,000			https://www.youtube.com/user/associationleucan	940		
Northern Ontario Families of Children with Cancer (NOFCC)	https://www.facebook.com/northernontariofamiliesofchildrenwithcancer/#	3,400										
Ontario Parents Advocating for Children with Cancer (OPACC)	https://www.facebook.com/OPACC	1,200	https://www.instagram.com/opaccorg/#	886			https://x.com/OpaccOrg	565	https://www.youtube.com/@opaccorg	52		
Pediatric Oncology Group of Ontario (POGO)	https://www.facebook.com/PediatricOncologyGroupofOntario	4,500	https://www.instagram.com/pogo4ki	2,186	https://www.linkedin.com/company/pediatriconcologygroupofontario/	3,000	https://x.com/POGO4kids	1,184	https://www.youtube.com/channel/UCf0DR6HFGOhoUX6	869		

			ds/?hl=en						sLt7g67A			
Sophia Smiles Pediatric Cancer Foundation	https://www.facebook.com/SophiaSmilesPCF/	434	https://www.instagram.com/sophiasmiles_/?hl=en	45								
Tali's Fund	https://www.facebook.com/talisfund/	5,300	https://www.instagram.com/talisfund/	1,462	https://www.linkedin.com/company/tali-s-fund/	299			https://www.youtube.com/channel/UCOwpLjd7Brxs7W5tPapVH-g	13		

On The Tip Of The Toes	https://www.facebook.com/FondationSurLaPointeDesPieds/	8,200	https://www.instagram.com/fondationsurlapointedespieds/	1,356	https://www.linkedin.com/company/fondation-sur-la-pointe-des-pieds/	656			https://www.youtube.com/channel/UCJMYilkAZGkoyKC1XTgfQlw	122	https://www.flickr.com/photos/pointedespieds/albums/	108
The Voboc Foundation	https://www.facebook.com/VobocFoundation/	1,500	https://www.instagram.com/voboc/?hl=en	552	https://www.linkedin.com/company/voboc/	646			https://www.youtube.com/user/VOBOCorg	10		
West Coast Kids Cancer Foundation	https://www.facebook.com/WestCoastKidsFd/	1,200	https://www.instagram.com/westcoastkidsfd/	1,339	https://www.linkedin.com/company/west-coast-kids-cancer-foundation/?originalSubdomain=ca	486						

Young Adult Cancer Canada	https://www.facebook.com/YoungAdultCancerCanada	6,700	https://www.instagram.com/yacancercanada/	2,886			https://x.com/yacancercanada	3,464	https://www.youtube.com/youngadultcancer	327		
Zippaport	https://www.facebook.com/zippaportshirts	573	https://www.instagram.com/zippaport/?hl=en	860								

Retrieved June and July of 2025.
